# Forces exerted during exercises by patients with adolescent idiopathic scoliosis wearing fiberglass braces

**DOI:** 10.1186/1748-7161-1-12

**Published:** 2006-07-21

**Authors:** Michele Romano, Roberta Carabalona, Silvia Petrilli, Paolo Sibilla, Stefano Negrini

**Affiliations:** 1 ISICO (Italian Scientific Spine Institute), Via Carlo Crivelli 20, 20122, Milan, Italy; 2 Don Carlo Gnocchi Foundation ONLUS, Care and Research Institute, Via Capecelatro 66, 20148 Milan, Italy

## Abstract

**Objective:**

To quantify and compare the forces exerted by scoliosis patients in fiberglass braces during exercises usually prescribed in departments where casts are made. The exercises are intended to increase corrective forces, activate muscles, stimulate ventilation and help the patient psychologically.

**Setting:**

Outpatient care.

**Patients:**

17 consecutive adolescent patients wearing fiberglass brace for idiopathic scoliosis.

**Interventions:**

Exercises (kyphotization, rotation, "escape from the pad") in different positions (sitting, supine, on all fours).

**Main outcome measure:**

Pressure detected by the F-Socket System between the rib hump and the pad of the brace.

**Results:**

In static and dynamic conditions, the position adopted did not alter the total pressure exerted by the brace, although the part of the sensor stimulated did vary. Kyphotization and rotation exercises produced a significant increase of pressure (+ 58.9% and +29.8%, respectively); however, the "escape from the pad" exercise, despite its name, did not produce any significant variation of pressure.

**Conclusion:**

Exercises in the brace allow adjunctive forces to be applied on soft tissues and through them, presumably on the spine. Different exercises can be chosen to obtain different actions. Physical exercises and sporting activities are useful in mechanical terms, although other important actions should not be overlooked.

## Background

Physical exercises are prescribed in some institutions for patients that wear braces for adolescent idiopathic scoliosis [1-3], but there is no scientific evidence for this practice [4,5].

The possible useful effects of an exercise regimen for a patient with adolescent idiopathic scoliosis wearing a corrective brace have been divided theoretically into the two categories of general and specific effects [6,3,11]. The former includes all beneficial modifications (obtained through the activation of muscles, the stimulation of ventilatory exchanges and psychological help) that physical activity provides the patient, while reducing the impairments and disabilities induced by wearing the orthosis.

Let us look at these singly:

1. Activation of muscles

In braced patients, it is normally thought that the supporting action of trunk muscles is reduced [9,10,12-15]. Exercises are proposed to:

a. avoid this effect, that could be more pronounced in adolescent patients using braces all day long;

b. have the effect of stabilizing the spine when the brace is removed.

2. Stimulation of ventilatory exchanges

Vital capacity and VO2 max are often reduced in patients whose scoliotic curvatures exceed 30 degrees Cobb [16,17]. The VO2 max is usually reduced beyond a level that might be explained by a decrease in vital capacity alone. The reduction has been attributed to lack of physical exercise [18,19]. Exercises are proposed to:

a. increase vital capacity;

b. train the patient so that both the cardiovascular and the musculoskeletal systems have an increased capacity to use oxygen;

c. improve respiratory ability from a neuromuscular standpoint.

3. Psychological help

Braces may induce a "negative body image" [20] in a growing child, that could, in turn, lead to an immature personality in adulthood. Exercises are proposed to reduce the psychological disability induced by the brace (which is not as great as that induced by the impairment itself), which may include a feeling of inferiority of the patient with respect to his/her friends.

Conversely, the category of specific effects relates to the pressure that braces exert on the spine through the soft tissues. Specific exercises have been proposed by Stagnara [3,2] and many others [6,7,13,10,12,13,15,21,22] with the rationale of increasing the corrective forces applied by the brace – somehow using movements as "dynamic tools" to amplify the corrective "static" forces applied by the orthosis.

Obviously such movements are instantaneous, but the immobilization of the ribs and spine that they induce (the former having modelling, and the latter, derotatory and deflective effects), could, in time and with repetition, play a major role in bringing about a positive effect of the brace [3,2].

Moreover, it is necessary to consider the following assumptions:

1. According to many etiological theories, the central nervous system could play an important role in the origin of the deformity [23,24];

2. It has been supposed that the soft tissues are not able to withstand passively the forces that should be applied by a brace in order to correct a scoliosis [25];

3. The brace corrective effect with respect to Cobb angle reduction is strongly correlated with the pressure exerted by the pads [26];

4. The strap tension should be set as high as possible for right thoracic curvatures [27];

5. Muscular contraction has been supposed to play a major role in the effect of braces [25,28]. Esthesio- and proprioceptive stimuli have also been considered important in bringing about a rearrangement of the postural system [1,23];

6. The electronic pressure sensor may be the best way to quantify the effectiveness of brace pad placement [29].

On the basis of these hypotheses, the forces applied during specific exercises and physical activities are important not only from a biomechanical standpoint, but also from a neurological perspective that aims to help the patient to develop a new spinal behavior. Thus, exercises that dually act on the spine – both to increase the forces of the brace and to drive vertebrae in the direction of the correction by means of the "escape from the pad" movement [3,30] – could be extremely useful.

Focusing on the specific effect of physical activity, the aims of our study were:

1. to ascertain the presence of muscle action and, if present, determine its importance;

2. to evaluate the theoretical possibility of developing a best "corrective exercise" in a fiberglass brace (in terms of forces exerted during the movement); and

3. to compare the exercises most commonly prescribed to achieve specific effects, such as kyphotization, rotation [9,10,3] and "escape from the pad" [3,30].

## Materials and methods

### Subjects

We examined 17 patients consecutively admitted to Don Gnocchi Foundation Orthopaedic Department to undergo treatment for idiopathic scoliosis. This treatment was based on two or three (depending on the degrees Cobb of the curvature) fiberglass non-removable braces (modified Risser type) [1]. The inclusion criteria were:

1. a diagnosis of adolescent idiopathic scoliosis;

2. the presence of a right thoracic or thoracolumbar curvature;

3. no significant pain during movements in brace;

4. second or third Risser fiberglass brace (so that, before the start of our evaluation, the patients involved in the study were accustomed to movements inside the brace); and

5. good execution of the proposed movements, as judged by a single, well-qualified physical therapist (MR).

Fourteen female and three male patients were included in the study. Age ranged from 12 to 17 years; curvatures from 36 to 52 degrees Cobb; 13 of 17 patients had double major curvatures and all patients had classic idiopathic right thoracic, left lumbar curvature patterns.

### Instrument

A pressure distribution measurement system (F-Scan System™- Tekscan, Inc. Boston, MA 02210 USA) was utilized which detects pressure by means of a sensor and handles data through a dedicated personal computer and software. The sensor is a rectangular plastic sheet 7.62 cm wide, 20.32 cm long and 0.1016 mm thick. It is a resistive-based device made up of a conductive paste interposed between two insulating sheets. The paste is applied so as to form a 6 × 16 cell grid on one side and a 6 × 4 cell grid on the other. The cells are connected to one another by conductive wires which guide the variation of resistance from the pressure applied to the device for measurement. The sensor is connected to a personal computer by an A/D unit able to control the scanning process. Important characteristics of the software include its ease of use and the capability of viewing the acquisition process in real time.

The repeatability of the F-Scan system has been verified in other applications [31,32]. We have already studied the capacity of the system to evaluate the forces applied by Fiberglass orthoses, and revealed how certain parameters influence data acquisition [32]. The most important of these are:

1. the size of the forces to be measured;

2. the temperature during measurements;

3. the time taken to perform measurements;

4. the interval between two consecutive measurements; and

5. sensor wear and tear.

The foregoing perturbations can be limited by calibrating the sensor carefully before the start of data acquisition. For comparison with the data obtained *in vivo*, a measurement is required that must be calculated when all the parameters are known and controlled [32].

### Brace

The Risser cast has a long tradition in scoliosis treatment [33,34] and is still used in some scoliosis centers around the world[1]. The brace utilized in this study was modified by using a different material (fiberglass) to avoid the last (third) phase of manufacturing [1,35]. The patient lays supine on a modified Risser bed, in traction. The following corrective actions are performed on the patient's trunk:

• passive deflection;

• derotation with a tissue band; and

• direct push on the rib hump by two pads that exert a strong pressure through a screw mechanism.

The brace is modelled directly on the patient's trunk with fiberglass bands. Two windows are cut to allow for expansion of the concave aspect of the scoliotic deformity once the material hardens.

### Movements evaluated

We evaluated those movements which can be performed in a seated position and which were thought to be capable of varying the corrective forces exerted on the scoliotic curvatures and rib humps by the brace [9,10,3]. The movements could either enhance or reduce the pressure of the pad. The following movements were tested:

1. kyphotization: a generalized flexion of the whole dorsal spine;

2. rotation against the pad of the brace: a movement of the apical aspect of the curvature against the pad of the brace;

3. "escape from the pad" movement [3,30]: a movement of the apical aspect of the curvature in the same direction as that of the passive corrective forces (*i.e*., away from the pad of the brace). The movement is proposed as a means of obtaining an active alignment of the spine.

The way the movements were performed are detailed below.

#### Kyphotization in sitting position (Figure 1)

**Figure 1:**
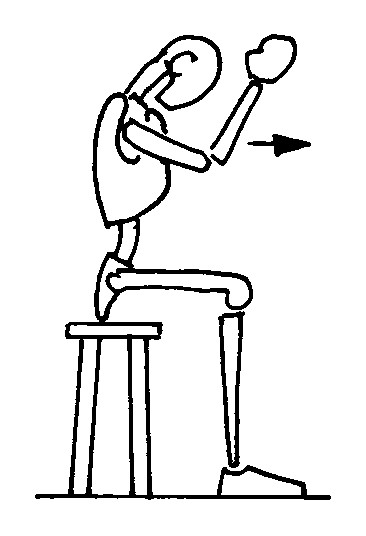
kyphotization in sitting position; The figures represent only the movement performed by the patient; the brace is not depicted in order to reveal the movement performed inside. The Risser cast brace pushes on the rib hump of the patients as well as laterally, while leaving a window on the opposite side.

a. the patient is seated;

b. the arms and elbows are flexed at 90°;

c. the elbows are pushed forward.

#### On all four kyphotization (Figure 2)

**Figure 2:**
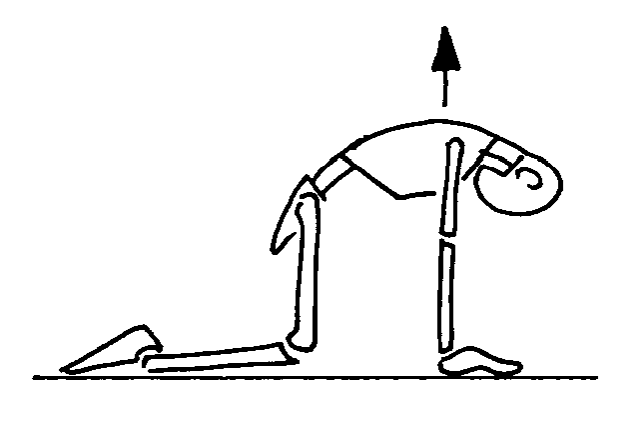
kyphotization on all fours; The figures represent only the movement performed by the patient; the brace is not depicted in order to reveal the movement performed inside. The Risser cast brace pushes on the rib hump of the patients as well as laterally, while leaving a window on the opposite side.

a. the patient is positioned on all fours;

b. trunk is flexed.

#### Rotation (simple) (Figure 3)

**Figure 3:**
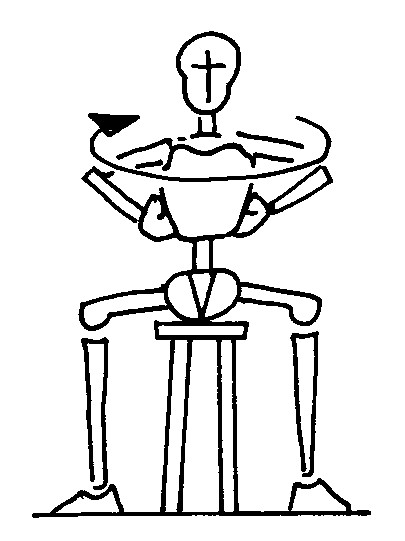
rotation (simple); The figures represent only the movement performed by the patient; the brace is not depicted in order to reveal the movement performed inside. The Risser cast brace pushes on the rib hump of the patients as well as laterally, while leaving a window on the opposite side.

a. the patient is seated;

b. the trunk is rotated against the pad.

#### Rotation against a wall (Figure 4)

**Figure 4:**
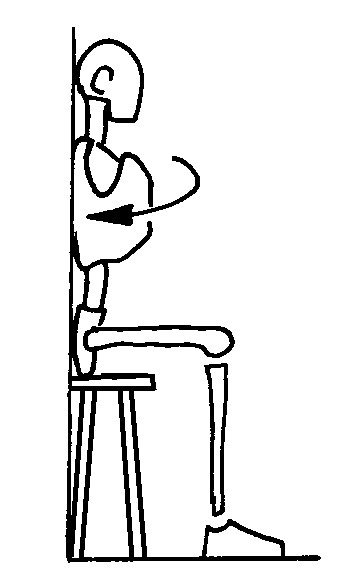
rotation against the wall; The figures represent only the movement performed by the patient; the brace is not depicted in order to reveal the movement performed inside. The Risser cast brace pushes on the rib hump of the patients as well as laterally, while leaving a window on the opposite side.

a. the patient is seated near a wall;

b. the trunk is rotated against the pad.

#### "Escape from the pad" (Figure 5)

**Figure 5:**
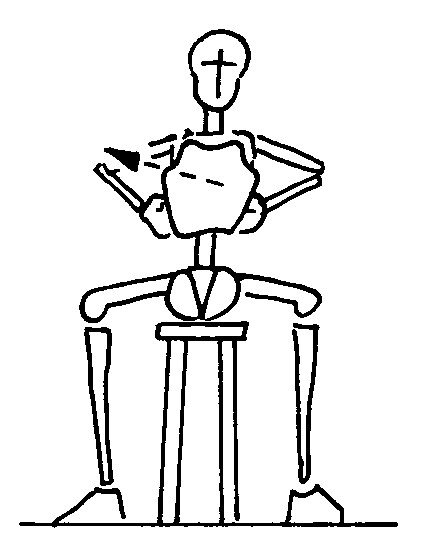
"escape from the pad". The figures represent only the movement performed by the patient; the brace is not depicted in order to reveal the movement performed inside. The Risser cast brace pushes on the rib hump of the patients as well as laterally, while leaving a window on the opposite side.

a. the patient is seated;

b. the rib hump is detached as far as possible from the pad.

#### Reference positions

For reference, we also evaluated the pressure values obtained at rest in the seated, supine and "on all fours" positions.

### Methodology

During the making of the fiberglass brace, the sensor is planted between the right (thoracic or thoracolumbar) rib hump and the pad, almost parallel to the spine. Twenty-four hours after the brace is made, the physical therapist reminds the patient of the movements required and watches to see that they are executed correctly. At this point, the pressure data is collected. At the end of the data acquisition process the sensor is removed.

Each recording lasts ten seconds and has a frequency of five frames per second. Data was collected following the sequence outlined below:

1. neutral position;

2. kyphotization;

3. simple rotation;

4. rotation against the wall;

5. "escape from the pad".

### Analysis of data

The results (pressure values and area stimulated) refer to the average of the 10 frames in which the maximum pressure on the sensor was obtained. The stimulated area was calculated by multiplying N, *i.e*., the number of activated cells (those whose value is over zero), by β = 1.6129 (the area in cm2 of a single cell). The pressure value corresponds to the average of the measurements obtained over the whole stimulated area of the sensor. The results refer to the full sensor area and data were analyzed using Excel 5.0, Statgraphics Plus 3.1 and Matlab 5.0 for Windows. Statistical analysis was nonparametric, due to the non-Gaussian distribution of the data (Shapiro-Wilks W test). Significance was reached at p < 0.05. The tests used for statistical analysis were the Wilcoxon [36] test, the Friedman [36] test for repeated measures and a multiple comparison procedure [37].

## Results

### Corrective pressure: absolute values

In the reference positions, the average pressure values applied by the fiberglass brace itself were as follows:

1. seated: 264.3 g/cm2 (range 191–436);

2. supine: 262.5 g/cm2 (range 142–367);

3. on all fours: 267.3 g/cm2 (range 186–381).

Given that the total surface area of the stimulated portion of the sensor averaged 105 cm2, it can be postulated that, without exerting any additional force through movements, there was an average overall force of 28 kg on the rib hump.

Through exercises, the force on the rib hump in the sitting position can be intensified as follows:

1. kyphotization: 47 kg;

2. simple rotation: 39 kg;

3. rotation against the wall: 40 kg;

4. "escape from the pad" movement: 32 kg.

The average pressure values during kyphotization in different positions are detailed below:

1. seated: 420 g/cm2 (range 303–958), increase in pressure of 58.9%;

2. supine: 423 g/cm2 (range 282–899), increase in pressure of 61.1%;

3. on all fours: 431 g/cm2 (range 283–882), increase in pressure of 61.2%.

With reference to the other exercises, the pressure values were lower:

1. simple rotation: 343 g/cm2 (range 238–809), increase in pressure of 29.8%;

2. rotation against the wall: 332 g/cm2 (range 222–797), increase in pressure of 25.6%;

3. "escape from the pad" movement: 309 g/cm2 (range 205–553), increase in pressure of 16.9%.

### Pressure

In the sitting position significant overall differences were found between exercises. When the sensor was divided in the latero-lateral direction, no differences emerged between exercises with the exception of kyphotization. When the sensor was divided in the cranio-caudal direction, differences emerged mainly in the proximal part of the pad. The central part registered increased pressure only during kyphotization, while no statistical differences emerged in relation to the distal part. In the sitting position, only the "escape from the pad" movement exercise failed to produce differences in relation to the reference position, while the biggest variations were found during kyphotization.

In the "on all fours" position, kyphotization nearly always increased pressure values on the pad; only in the proximal area did we fail to observe this difference

In the supine position there was an overall increase in pressure. When the sensor was divided in the cranio-caudal direction, an increase in pressure during the exercise was found in the central area, but no differences emerged in relation to the latero-lateral direction.

### Area

No statistically significant differences emerged between the area values during different exercises, with the exception of the paracostal and central values in the sitting position. (Submitted to the Friedmann test, it was not possible to ascertain which exercise might be able to determine such differences.)

### Reference positions

We found no statistically significant difference, in relation either to pressure values or area, when comparing the reference positions. This was also the case when the sensor was divided into different regions.

## Discussion

### Reference positions

The absence of differences in pressure values applied by the fiberglass brace on the rib hump in different positions is interesting. In fact, we could interpret the three reference positions in relation to the forces which act passively on the brace-body-spine system. In the sitting position, the action of the force of gravity compresses the spine from above, increasing the kyphosis and pushing the posterior part of the trunk toward the pad. In the supine position, the brace is compressed between the body and the ground surface, while in the"on all fours" position there is no such compression. The differences among these forces did not influence the force administered by the brace itself.

### Different exercises

All exercises done to increase the forces between the rib hump and the brace increased the pressure significantly as expected. The increase in pressure reached a doubling in many cases. There was no variation in the area stimulated, which means that the area depends on something different from exercise that is assumed to be determined during the making of the brace (presumably the shape of the body and the pad, and/or the interaction between them).

In terms of pressure exerted, the type of exercise done was more important than the position or way in which the exercise was performed. All kyphotization movements were more effective in terms of the amount of pressure exerted than the rotation movements. The more pronounced effect of the kyphotization as compared to the rotation exercises was surprising, as rotation was thought to be a more specific movement against the pad. Our results can presumably be explained by the strength of the muscles involved (flexors versus rotators of the spine) and by the movement itself (easier in sagittal than in the horizontal plane).

No statistically significant difference was found between the neutral reference position and the "escape from the pad" exercise – a finding which was most peculiar and completely discordant with the supposed aim of the exercise [3]. Moreover, the absolute values of pressure were on average found to be higher in the "escape" position than in reference position. A partial explanation for this unexpected phenomenon is that the exercises were performed the day after the making of the second/third fiberglass brace of the sequence – a stage at which it is not easy to detach the skin from the pad. Presumably, the same study performed at a different moment in time would produce different results. Even though it was not found to be useful in term of forces, the "escape from the pad" movement may nevertheless be a helpful way of indicating to the central nervous system the direction in which the spine should go in order to correct the deformity.

The described exercises must be considered only with respect to wearing braces like the one proposed in this paper (with a direct push on the rib hump). While the three-dimensional combination of planar movements inside the brace may produce positive results, kyphotization without the constraint of the brace in the frontal and horizontal planes could result in a rib hump increase.

### Different positions during kyphotization

Kyphotization was the only exercise analyzed in all positions. It always revealed an increase in pressure – a finding which also emerged when considering the latero-lateral division of the sensor.

The analysis of the cranio-caudal division was interesting, as differences were found in the behavior of the spine during the same exercise. There appears to be, in the sitting position, an extension of the distal part of the spine with a correspondent increase of pressure on only the proximal part of the sensor, while in the "on all fours" position the phenomenon is reversed, with an increase in pressure evidenced in only the distal part of the sensor. In both of these situations, the brace lacked the external support provided by the ground in the supine position; indeed in supine, kyphotization increased pressures only in the central part of the pad.

### Rotation and "escape from the pad"

There were no differences between the two types of rotation exercises; the presence or absence of the support of the wall was not significant, nor was the behavior of the spine with regard to the division of the sensor into different areas as previously described.

Even though no significant differences emerged, an analysis of the behavior of the forces on different parts of the pad during the "escape from the pad" movement suggested that the action produced here was simply an extension of the spine, with a decrease of pressure in the distal region of the sensor. It is possible that the patient was unable to follow and extend the action of the brace actively while constrained inside the fiberglass brace utilized; a different result might have emerged from a study utilizing a different type of brace, such as the Chêneau 2000 brace [38-40] that incorporates empty chambers to allow for greater spinal mobility into targeted regions. Although the brace used in this study is thought to be comparable in its shaping action to braces built in the Chêneau 2000 mode, the relative ability of the patient to actively adopt the corrective posture within the brace can perhaps best be assessed by the utilization of the electronic pressor sensor method.

This study was performed in 1996 and has not been published previously. Although the Risser braces are no longer in use by the authors, new concepts of treatment have since been developed [41] that continue to apply forces on the rib humps in different ways. Through the indirect measurement of forces applied to the spine, the utilization of the electronic pressure sensor method presents an opportunity to assess the effectiveness of new treatments.

### Clinical and future research implications

We recognize that today there is no standard of bracing for scoliosis and that different schools are competing and comparing notes. The Chêneau and Rigo [38-40,42], Risser or Lyon [1,2] and SPoRT [41] schools propose different concepts of correction. The exercises investigated herein are rooted in all three groups and follow their theoretical approaches. The results of this study indicate that therapeutic exercises can increase the forces that are produced by the brace and may thereby help to increase the corrective action of the orthosis.

This study was performed in the first 24 hours of bracing, and the results could change in the long term (such being one of the reasons why braces are renewed). Future research could address the variations of these effects over time.

Exercises and exercise positions adopted can be selected depending on the desired action on the spine. On the basis of our experience and on the minor pressure differences found, we suggest varying the stimuli on the trunk of the patient. Varying the truncal stimuli would be expected to produce not only a better biomechanical action on the spine, but also a better neurological stimulation of the brain to elicit motor learning. The latter consideration may not apply in cases of severe rib hump or when a greater aesthetic improvement is required, as the reduction of the rib hump is primarily a mechanical matter.

To our knowledge, this is the first study showing the effects of physical exercises in orthosis on idiopathic scoliosis. Braces are effective in idiopathic scoliosis treatment [4,43-45], and thus the search for the most useful way of helping patients when they wear braces must be intensified. Physical exercises could serve not only to reduce the secondary effects of braces (muscular, respiratory and psychological impairment) but also in an attempt to improve the action of the orthosis itself [3,46,47].

Future research could address the same topic in classic plastic braces or in traditional casts, and in cases of less severe curvatures in which applied forces may be less significant. Our results suggest that exercises may prove helpful in these cases as well.

## Conclusion

We are able to conclude that:

1. in static conditions, the position of the patient does not interfere with the forces exerted by the fiberglass brace;

2. all exercises produce a significant increase in the mechanical forces exerted at rest by the fiberglass brace, with the exception of the"escape from the pad" movement, whose action should be exactly the opposite;

3. the "escape from the pad" movement inside the fiberglass brace used is not so; it was not possible to demonstrate any decrease of pressure on the pad;

4. although the "escape from the pad" exercise does not represent an actual mechanical "escape from the pad" movement inside of the fiberglass brace, this is apparently how it is perceived mentally, as evidenced by the extension movement observed;

5. the strongest mechanical forces are produced by kyphotization exercises;

6. rotation exercises are mechanically less efficient than kyphotization; and

7. through kyphotization exercises it is possible to select different localized actions on the spine, depending on the patient's requirements.

The method we used was found to be a useful instrument for evaluating forces exerted during exercises performed in the fiberglass brace. Although a common aim during exercises of this type is to obtain maximum corrective forces, it is also fundamentally important to remember that physical activity plays an important role in the prevention and reduction of impairments and disabilities – a role that extends beyond the mere exertion of mechanical forces. For this reason, it is important for patients to exercise in their brace, to continue practicing sports and to be encouraged to continue exercising, in and outside of school.

Since this study, kyphotization exercises have become a welcome addition to the in-brace SEAS (Scientific Exercises Approach to Scoliosis) protocol, while the already established recommendation of sports activities in and out of the brace has been maintained.
